# Epiploic Appendagitis: A Commonly Overlooked Differential of Acute Abdominal Pain

**DOI:** 10.7759/cureus.12807

**Published:** 2021-01-20

**Authors:** Jude-Theddeus E Akubudike, Oghenerukevwe F Egigba, Badri Kobalava

**Affiliations:** 1 Surgery, New Vision University, Tbilisi, GEO; 2 General Surgery, Aversi Clinic, Tbilisi, GEO

**Keywords:** epiploic appendagitis, appendicitis, acute abdominal pain, misdiagnosis, diverticulitis

## Abstract

Epiploic appendagitis is an unusual and very commonly overlooked source of acute abdominal pain. Its incidence is highest in middle-aged obese males. It presents clinically as a focal lower quadrant abdominal pain, usually in the absence of pyrexia, nausea, vomiting or change in bowel habit, and unremarkable laboratory markers. Due to its vague presentation, epiploic appendagitis may be mistaken for other more severe causes of acute abdominal pain like diverticulitis and appendicitis, thereby causing patients to undergo unwarranted management interventions and hospital stay. Epiploic appendagitis is usually diagnosed through imaging, most commonly computed tomography (CT). This condition is largely self-resolving and can be managed conservatively with nonsteroidal anti-inflammatory drugs (NSAIDs). Operative intervention is usually employed when symptoms persist or when complications arise. We present a case of epiploic appendagitis in a patient who presented with right lower quadrant pain initially misdiagnosed as acute appendicitis.

## Introduction

Epiploic appendagitis is a rare, benign inflammatory process [1] arising as a result of the twisting of an epiploic appendage, a pedunculated fat-filled peritoneal out-pouching, or thrombosis of its draining vein thereby leading to the ischemic necrosis and subsequent inflammation of the affected appendage [2]. It may also occur secondary to other inflammatory conditions affecting adjacent abdominopelvic organs such as appendicitis, diverticulitis, cholecystitis, pancreatitis, and salpingitis [3,4].

Epiploic appendagitis has shown increased prevalence in the male and obese population. Other factors associated with this condition are strenuous exercise and the presence of an abdominal hernia [2].

Diagnosis is made through imaging, with classic computed tomography (CT) findings of hyperaenuating ring sign, central dot sign, and mild thickening of the bowel wall. Treatment is usually conservative with nonsteroidal anti-inflammatory drugs (NSAIDs) and symptoms resolving within few days [5].

This article was previously presented as a poster at the 2021 Craiova International Medical Students’ Conference on November 20, 2020.

## Case presentation

A 57-year-old man with no significant past medical or surgical history presented to the emergency department complaining of pain in the right lower quadrant that started a day prior to presentation. He described the pain as sharp and sudden in onset, and was initially felt in the mid-abdomen but worsened and migrated to the right lower quadrant on the day of presentation. He denied any associated fever, chills, vomiting, diarrhea, dysuria, hematuria, or other gastrointestinal symptoms.

Vital signs and laboratory analysis were unremarkable. On physical examination, the patient was not in acute distress, and abdominal exam showed tenderness in the right iliac fossa along with guarding.

**Figure 1:**
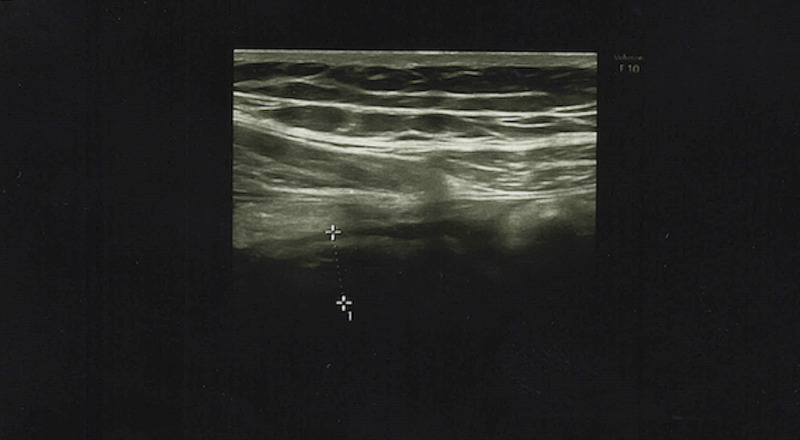
Ultrasound showing fluid in the ileocecal fossa and a 10 mm appendiceal diameter with periappendiceal inflammation

Ultrasonography of the abdomen revealed fluid in the ileocecal fossa, a 10 mm appendiceal diameter with periappendiceal inflammation, which was suggestive of acute appendicitis (Figure 1).

**Figure 2:**
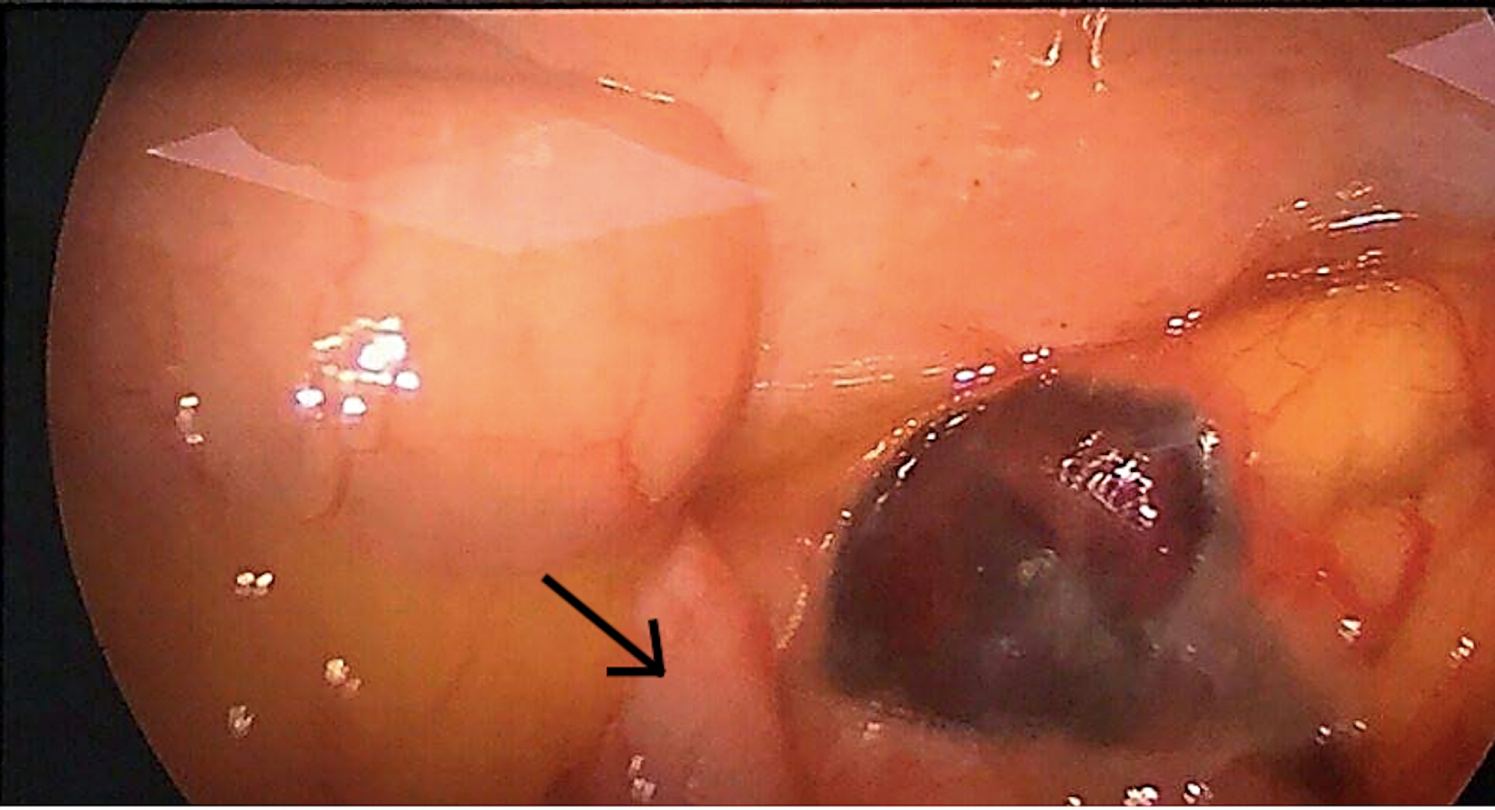
Arrow showing laparoscopic view of normal appearing appendix.

**Figure 3:**
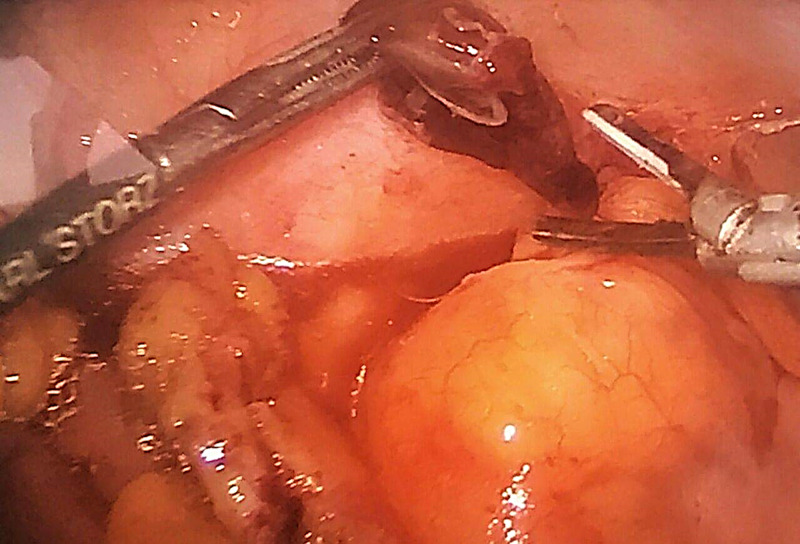
Intraoperative view of necrotic epiploic appendage before resection.

Consequently, laparoscopic appendectomy was scheduled. Intraoperatively, a moderate amount of serous fluid in the right iliac fossa and Douglas pouch was found and aspirated. The appendix did not appear to be inflamed (Figure 2). A necrotic epiploic appendage of approximately 20 x 20 mm in size, adjacent to the cecum (Figure 3), was visualized and resected along with the appendix. Thereafter, a post-operative diagnosis of epiploic appendagitis was established. The patient was managed with intravenous fluids, as well as analgesics and antibiotics, for prophylaxis against post-surgical infections, and was discharged on post-operative day three.

## Discussion

Epiploic appendages, also referred to as omental appendages, first described anatomically by Vesalius in 1543 [5], are 50-100 pedunculated fatty structures (0.5-5 cm in length) arising from the anti-mesenteric serosal surface of the colon. These structures are attached by a vascular stalk and arranged in two separate rows next to the anterior and posterior tenia coli over the superficial region of the colon, except for the transverse colon, where only a single row is present because of the attachment of the greater omentum to the tenia omentalis, and a complete absence in the rectum [2,6]. Its vascular stalk comprises one or two small circular arteries arising from the vasa recta longa of the large intestine, and a tortuous vein that courses through a narrow pedicle for venous drainage [3,7]. The limited vascular supply of these structures, alongside their pedunculated morphology and enhanced mobility, increases the risk of torsion with ensuing ischemia or hemorrhage [3]. The actual function of omental appendages is not known, although it has been suggested by some to serve as a reservoir for blood, assist in colonic absorption, and provide cushion and immunity against pathogens [1,5].

In prior literature, the reported incidence rate of epiploic appendagitis was about 8.8 cases/10 million population/year [2,5], accounting for about 1.3% of abdominal pain presentations to the emergency department [4], although this may be an underestimation as this condition is commonly misdiagnosed. It is predominantly diagnosed during the second to fifth decades of life, with a prevalence four times more in males than females [3], and very rarely seen in children [4]. 

Epiploic appendagitis most commonly presents as an insidious non-migratory lower abdominal pain that is exacerbated by coughing and abdominal stretching. Pyrexia, nausea, vomiting, or change in bowel habits are very rarely seen with epiploic appendagitis [1,2,5]. The preponderance of cases involves the left lower quadrant due to the high frequency of sigmoid colon appendage involvement [1], although it can be sometimes experienced on the right, like in our case. The principal causes of epiploic appendagitis are torsion and inflammation [1]. Although disputed by some, epiploic appendagitis is also reported to be associated with obesity, hernia incarceration, intestinal obstruction, and exercise injuries [2]. In a seven-year retrospective case control study involving patients with epiploic appendagitis and patients with other causes of acute abdomen, patients with epiploic appendagitis were discovered to have 60% more abdominal adipose volume, 117% greater visceral fat area, and 35% more subcutaneous fat than the other subgroup with acute abdomen [8].

A diagnostic hypothesis based solely on clinical findings often does not permit the recognition of this condition [4]. Considering the lack of characteristic signs and symptoms, epiploic appendagitis, clinically, is a diagnosis of exclusion [9]. Physical examination usually reveals a soft, non-distended abdomen and localized rebound tenderness on palpation with or without a palpable mass [1-3,5] and possible guarding [4]. Laboratory findings in patients with epiploic appendagitis usually fall within the normal range and any abnormalities, if present, are mostly non-diagnostic.

Historically, epiploic appendagitis was most commonly diagnosed during exploratory laparotomy/laparoscopy, but in 1986 the imaging features for the condition were identified and described [7]. The most preferred form of imaging for diagnosis of epiploic appendagitis is contrast-enhanced computed tomography (CT), with more than 90% sensitivity and specificity [6]. The hallmark findings on CT imaging include fat-density ovoid lesion (hyperattenuating ring sign), mild bowel wall thickening, and a central high-attenuating focus within the fatty lesion (central dot sign) [2,5]. Although symptoms associated with epiploic appendagitis typically resolve within a few days to a few weeks after onset, classic CT features may remain for up to six months [1,2]. Also, calcifications may be seen on follow-up studies and usually represent old infarcts [10]. Besides CT, other imaging modalities used in evaluating epiploic appendagitis, especially in pregnancy and pediatric populations are ultrasound, and less frequently, magnetic resonance imaging (MRI) [7] due to its non-availability in many emergency settings [11], especially in third world countries. Ultrasound findings usually show an oval hyperechoic, non-compressible mass adjacent to the colonic surface with absence of central blood flow [2,7]. MRI findings include the presence of an oval mass with fat tissue signal intensity in T1 and T2 weighted MRI images and ring enhancement in contrast agent (gadolinium) enhanced T1 weighted imaging [2,6,7].

Untreated, a variety of complications may result. Adhesions may develop due to inflammation in the surrounding tissue, thereby causing even more severe symptoms. Other possible complications are peritonitis, intestinal obstruction, local abscess formation, and intussusception [12].

Common differentials of epiploic appendagitis include acute diverticulitis, omental infarction, acute appendicitis, sclerosing mesenteritis, and less commonly, acute cholecystitis, salpingitis, ectopic pregnancy, ovarian cyst rupture, and ovarian torsion [1].

Epiploic appendagitis is typically regarded as a self-resolving condition and can be managed conservatively with NSAIDs [2,13,14]. Antibiotics are not indicated in management of epiploic appendagitis as many reports failed to show positive benefits regarded to their use [7,11]. Laparoscopic excision of the inflamed appendage is indicated if conservative management fails to eradicate symptoms [5,7,14] or in patients with complications requiring operative management [7]. Although epiploic appendagitis has a high incidence of abdominal pain, it generally has an excellent prognosis with no published records of mortality. Recurrence also is rare, but has been reported in cases managed conservatively [15].

## Conclusions

Epiploic appendagitis is an underdiagnosed condition in patients presenting with acute abdominal pain. The increased use of CT and ultrasound for diagnosis has caused a reduction in its misdiagnosis. Conservative management is preferred, with surgical intervention reserved for cases of recurrence or complications. This case report highlights the importance of its inclusion as a differential for acute abdominal pain.
